# Literary Note

**Published:** 1907-07

**Authors:** 


### LITERARY NOTE.

THE special June number of the *Annals of Surgery* received notice in this department of our June issue. The July number of the *Annals* will also be a special issue containing seven papers on "End Results Following Operation for Carcinoma of the Breast," which were discussed at the recent meeting of the American Surgical Association at Washington. Among these will be a remarkable paper by Dr. Halsted of Johns Hopkins Hospital. There will also be eight other valuable contributions by eminent specialists in addition to the reports of the transactions of the New York Surgical Society.

